# Interleukin-22 and acute pancreatitis: A review

**DOI:** 10.1097/MD.0000000000035695

**Published:** 2023-11-03

**Authors:** Xinjuan Fu, Zhigang Xiu, Hongwei Xu

**Affiliations:** a Department of Gastroenterology, Shandong Provincial Hospital, Shandong University, Jinan, China; b Gastroenterology center, Qingdao Hiser Hospital Affiliated to Qingdao University (Qingdao Traditional Chinese Medicine Hospital), Qingdao, China; c Department of Gastroenterology, Shandong Provincial Hospital Affiliated to Shandong First Medical University, Jinan, China.

**Keywords:** acute pancreatitis, autophagy, interleukin-22, signal transducer and activator of transcription 3

## Abstract

Acute pancreatitis (AP) is one of the most common gastrointestinal diseases, and it is divided into 3 types according to its severity:mild acute pancreatitis, moderately severe acute pancreatitis, and severe acute pancreatitis. The mortality in severe acute pancreatitis is approximately 15% to 30% due to multiorgan dysfunction and the lack of specific treatment. Interleukin-22 (IL-22) is a member of the Interleukin-10 family, and it can activate several downstream signaling pathways by binding to its receptor complex, thus it is involved in cell differentiation, proliferation, and apoptosis. Some studies have reported the elevated level of IL-22 in patients with AP, which suggests IL-22 may be involved in the pathogenesis of AP. And many studies have shown that IL-22 had a protective effect against AP. This article reviews the characteristics and mechanism of IL-22 and its role in AP to provide insight into the treatment of AP.

## 1. Introduction

There are various factors that can trigger acute pancreatitis (AP), including gallstones, alcohol abuse, hyperlipidemia, trauma, infections, medication, and so on. The initial event in AP is premature activation of zymogens, which stimulate inflammation and cellular injury via autocrine and paracrine signaling pathways. Interleukin-22 (IL-22), which is produced mainly by activated CD4^+^ T cells, acts by binding to a heterodimeric receptor (consisting of IL-22R1 and IL-10R2), then it activates several signaling pathways and upregulates some genes expression, and it plays a key role in antimicrobial defense, tissue repair, and inflammation.

In the pancreas, IL-22 has been shown to reduce acinar cell apoptosis, enhance acinar cell regeneration, and limit inflammation and fibrosis. Recent studies have reported that IL-22 level is elevated in patients with AP and experimental models of AP. Administration of exogenous IL-22 has been shown to ameliorate AP by reducing pancreatic inflammation and tissue damage, and improving survival rate. Further studies are needed to elucidate the precise mechanism of IL-22 in AP and to evaluate its potential value as a therapeutic target for the treatment of this complex and multifactorial disease.

## 2. The characteristic of IL-22

### 2.1. The source of IL-22

IL-22 is a member of the IL-10 cytokine family and is secreted by immune cells. It was first identified in IL-9 treated mouse lymphoma cells^[1]^ and was initially called IL-10-related T cell-derived inducible factor. Its protein is composed of 179 amino acids in mice and 146 amino acids in humans,^[2]^ with 79% homology between the 2 species.^[3]^ The major source of IL-22 is CD4^+^ T cells, including Th22 cells, Th1 cells and Th17 cells, with a small proportion coming from CD8^+^ T cells, natural killer cells, γδT cells, and innate lymphocytes. Additionally, non-lymphocyte cells such as fibroblasts, mast cells, macrophages, and neutrophils may also produce IL-22 in response to various pathological stimulus. It has been reported that mast cells have been identified as the major source of IL-22 in patients with dermatitis and psoriasis.^[4]^

IL-22 primarily acts on nonimmune cells expressing the homologous IL-22 receptor, which consists of IL-10R2 and IL-22R1 subunits and includes extracellular, transmembrane, and intracellular signaling regions. IL-10R2 is ubiquitous, whereas IL-22R1 is almost exclusively expressed in cells of non-hematopoietic origin, and its expression is abundant in the pancreas.^[5,6]^ IL-22R1 is also expressed in the skin, colon, small intestine, liver, and kidney. However, studies^[7]^ have reported that hematopoietic cells, especially circulating myeloid cells of primary Sjogren syndrome patients, also express IL-22R1, and IL-18 and IL-22R1 are correlated directly.

In addition to the IL-22 receptor, IL-22 binding protein (IL-22BP) is another receptor of IL-22, and IL-22BP is encoded by 231 amino acids. The extracellular structure of IL-22BP is resembles that of IL-22R1, and there is a 34% homology in amino acid sequences between them.^[8,9]^ However, IL-22BP lacks intracellular and transmembrane domains, and it is mainly expressed in lymph nodes, spleen, thymus, and the digestive system (such as the stomach, intestine, pancreas, and esophagus). IL-22BP is capable of neutralizing IL-22 activity, and its stable expression in tissues and organs can prevent the pathological increase of IL-22. As a soluble receptor with higher affinity to IL-22 than IL-22R1, IL-22BP can competently inhibit the formation of the IL-22-IL-22R1 complex, thereby affects the function of IL-22. Studies have demonstrated that IL-22BP has a pro-inflammatory effect by interacting with IL-22 and affecting epithelial barrier function.^[10]^

### 2.2. Biological function and mechanism of IL-22

IL-22 performs a range of functions by binding to its receptor complexes, including cell differentiation and mucosal defense and inflammatory, and its function varies by tissue and disease. IL-22 receptor complexes belong to the Class II cytokine receptor family. IL-22 binds to its receptor complexes and activates the classical Janus kinase 1)/Tyrosine kinase 2 signaling, subsequently activates the signal transducer and activator of transcription 3 (STAT3), along the mitogen-activated protein kinase pathway, and protein kinase B (AKT or PKB) is also activated.^[11–15]^ Mitogen-activated protein kinase pathway comprises extracellular regulatory protein kinases, kinase p38, and c-Jun N-terminal kinases. In addition, STAT1^[16]^ and STAT5 are also targets of IL-22 signaling. STAT3 signaling pathway is the primary signal pathway of IL-22 and its phosphorylation on the serine residue 727 is unique to interleukin.

IL-22 targets cells present in the skin, pancreas, liver, kidney, lungs, and other organs, and it plays significant roles in infections, tissue regeneration, tumors, and autoimmune diseases. A study shows the critical role of IL-22 in regulating intestinal cell homeostasis and promoting epithelial regeneration in inflammatory bowel disease.^[17]^ It is noteworthy that IL-22 promotes epithelial regeneration, innate defense, and membrane mucus production, with similar effects in healthy individuals and inflammatory bowel disease patients.^[18]^ Study^[19]^ in mice with necrotizing enteritis have shown that IL-22 can ameliorate intestinal inflammation by promoting epithelial regeneration. In the liver, IL-22 can regulate STAT3/ERK/Akt signaling pathways, then increases the anti-inflammatory/pro-inflammatory-Kupffer cells ratio, consequently delays the occurrence of liver fibrosis,^[20]^ and IL-22 alleviates acetaminophen-induced liver injury by enhancing autophagy,^[21]^ bacteria engineered to produce IL-22 reduced ethanol-induced steatohepatitis.^[22]^ During intestinal rotavirus infection, production of the IL-22 induced a protective gene expression program in intestinal epithelial cells.^[23]^ IL-22 exhibits a protective effect in ovalbumin-induced asthma mouse model by inhibiting the inflammatory cell infiltration and the airway hyperresponsiveness.^[24]^ Studies show that IL-22 alleviates acetaminophen-induced kidney injury by inhibiting mitochondrial dysfunction and inflammatory response.^[25]^ Additionally, IL-22 activates myocardial STAT3 signaling pathway and prevents myocardial ischemia-reperfusion injury.^[26]^

However, IL-22 has a dual function, it demonstrates pathogenicity effect besides its protective effect on tissues and organs. In psoriasis, the significantly elevated plasma IL-22 levels are correlated with the disease severity, and skin-infiltrated T and natural killer cells are the sources of IL-22 in psoriasis,^[27]^ while ultraviolet B facilitates skin inflammation by increasing keratinocyte’s responsiveness to IL-22,^[28]^ IL-22 aggravates lupus nephritis by promoting macrophage infiltration.^[29]^ The elevated expression of IL-22 is also involved in the occurrence and development of malignant tumors. Research has shown that IL-22 promoted the migration and invasion ability of oral squamous cells without increasing their viability.^[30]^ Another study revealed that IL-22 mediate chemotactic migration of breast cancer cells and macrophage infiltration of bone microenvironment, which achieved through enhancing sphingosine-1-phosphate/sphingosine-1-phosphate receptor signaling,^[31]^ and IL-22 promoted proliferation and invasion of osteosarcoma cells by activation of STAT3.^[32]^ Protopsaltis et al^[33]^ reported that IL-22 could induce endothelial cell proliferation, survival, chemotactic and neovascularization in choroid transplantation mice, and blocking IL-22 with antibodies could inhibit tumor angiogenesis, which suggesting that IL-22 maybe a potential therapeutic target for blocking tumor angiogenesis.

## 3. The mechanism of IL-22 in AP

### 3.1. Relationship between IL-22 and AP

AP is an inflammation characterized by pancreatic edema, hemorrhage, and necrosis, resulting from the self-digestion of pancreatic tissue due to various causes. Lipase, amylase, phospholipase, and the inflammatory mediators are involved in AP, with or without associated organ dysfunction. The mechanism of pancreatic enzyme activation, aggravating or protective factors are extensively studied. IL-22 is involved in various pancreatic disease processes, such as pancreatic inflammation, pancreatic fibrosis, pancreatic cancer. Research has demonstrated that serum IL-22 levels are significantly elevated in both human and animal models with AP, indicating the association of IL-22 in the occurrence and progression of AP.

A study^[34]^ found that the increased serum IL-22 levels in AP was not related to the severity of the disease, however, another study^[35]^ demonstrated that serum IL-22 levels in AP patients had a positive correlation with disease severity, further research is necessary. Xue et al^[36]^ suggested that IL-22 is involved in the pathogenesis of AP, and activation of the aromatic hydrocarbon receptor can induce the expression of IL-22, which has a protective effect on mice with AP.

### 3.2. Protective effect and mechanism of IL-22 in AP

Pancreatic acinar cells physiologically synthesize trypsinogen in an inactive form, pancreatic fluid enters the duodenum via the Oddi sphincter, and enterokinases secreted by duodenal mucosa can activate them. Abnormal activation of pancreatic enzymes leads to pancreatic acinar injury in AP, which involves the activation of inflammatory cascade, endoplasmic reticulum stress, and impaired autophagy.^[37,38]^ Some studies have shown that IL-22 can alleviate acinar cell damage in AP induced by caerulein. Additionally, activation of the Aryl hydrocarbon receptor protects mice from AP by inducing expression of IL-22.^[36]^ And administration of IL-22 before AP prevented the AP induction in a mild acute pancreatitis (MAP) model, and IL-22 knockout mice had less acinar cell injury but increased inflammatory cell infiltration.^[39]^ However, in severe acute pancreatitis (SAP) models, IL-22 administration during AP significantly ameliorated the pancreas injury. These researches suggested that IL-22 has protective effect on AP, but the specific mechanisms in MAP and SAP may be different. The specific mechanism of IL-22 in AP is unclear up to now, it may be related to STAT3, autophagy, PAP and so on, and we summarize as follows.

#### 3.2.1. Activating the STAT3 pathway.

AP animal model can be induced by caerulein, arginine or intraductal infusion of 5% taurocholic acid separately. Many studies have been conducted to explore the mechanism of IL-22 in patients with AP and AP mice model. IL-22 activates multiple signaling pathways through binding to receptors, and then performs important physiological functions. STAT3 is the predominant signaling pathway of IL-22, and it is known to be activated in acinar cells. STAT3 is activated by Janus kinase, and is involved in various cell physiological functions such as cell proliferation, apoptosis, and differentiation.

Research^[40]^ indicates that the STAT3/pancreatitis-associated protein 1 (PAP1) pathway has a protective effect on caerulein-induced AP by crossing STAT3 mice with Pdx1-promoter Cre transgenic mice. Moreover, IL-22 can ameliorate lung injury caused by arginine induced SAP through STAT3 pathway,^[41]^ and IL-22 protects intestinal mucosal barrier in SAP through decreasing colonic mucosal permeability, activating pSTAT3/Reg3 pathway, and restoring fecal microbiota abundance.^[42]^ It has also been reported^[43]^ that IL-22 enhanced the expression of antimicrobial peptides and antiapoptotic genes by activating STAT3 signaling pathway, and attenuated L-arginine-induced AP and intestinal mucosa injury in mice.

#### 3.2.2. Autophagy.

Autophagy is an evolutionally conserved cytoplasmic degradation process, and basal autophagy can maintain the physiological function of pancreatic acinar cell.^[44]^ Autophagy comprises 2 steps: the formation of autophagosomes and subsequent fusion of the autophagosome with lysosome to form an autolysosome.

Studies have shown that, due to the dysfunction of key organelle, autophagy is impaired in AP. Autophagy-lysosome fusion is affected, leading to the accumulation of autophagosomes, and excessive autophagy causes premature trypsinogen activation and acinus injury.^[45–48]^ However, inhibiting autophagy can relieve the AP severity,^[49]^ and autophagy regulation can ameliorate systemic organ injury and alter the progression of L-arginine or caerulein induced AP in mice.^[50]^ Studies have indicated that activating the nuclear factor-κB pathway enhances autophagy of pancreatic acinus cells in AP, and inhibiting this pathway decreases the severity of AP.^[51]^ Feng et al^[52]^ found that IL-22 inhibited the autophagy pathway and then ameliorated caerulein-induced AP by combining B-cell lymphoma-2(Bcl-2) and Bcl-XL with Beclin-1, and IL-22 could be a promising drug in the future for the treatment of pancreatitis.

#### 3.2.3. Others.

PAP is an acute phase protein produced during AP, comprising PAP2, PAP1, and PAP3, also known as Reg3α, Reg3β, and Reg3γ, respectively. They play an important role in apoptosis, regeneration, inflammation, immunity, etc. This secreted protein has a molecular weight of 16 kDa, which is undetectable in normal pancreatic tissues, but it is highly expressed in AP. A study has revealed that PAP can function as a protective agent by inhibiting local and systemic inflammation during AP.^[53]^ Studies on PAP/HIP knockout mice revealed that while acinar damage was reduced in caerulein-induced MAP, inflammatory infiltration was more severe. Conversely, administration of recombinant PAP/HIP to PAP/HIP (-/-) mice reduced pancreatic inflammation and apoptosis, activated STAT3 and enhanced suppression of cytokine signaling 3 levels.^[54]^ Another research has shown that the cerulein-induced reduction in cell viability and increase in apoptosis are reversed by overexpression of PAP-1 in PAP-1-transfected cells.^[55]^ Reg3 levels of IL-22-deficient mice were significantly reduced in caerulein-induced AP, and IL-22 might regulate Reg3 expression in acinar cells via STAT3.^[39]^

## 4. Conclusion

AP, especially SAP, is still a very challenging disease in clinical practice, and its etiology and mechanism were not very clear up to now. In this paper, we analyzed the biological characteristics of IL-22 and its mechanism in AP. Previous studies have shown that IL-22 has a protective effect on AP animals’ models, although the mechanism of MAP and SAP may not be the same. The possible mechanism of IL-22 in AP was investigated from STAT3, autophagy, and PAP. Further investigation of the mechanism of IL-22 in AP is necessary, and this may lead to more favorable outcomes for AP patients, particularly those with SAP.

## Author contributions

**Conceptualization:** Xinjuan Fu, Hongwei Xu.

**Formal analysis:** Xinjuan Fu, Zhigang Xiu, Hongwei Xu.

**Funding acquisition:** Hongwei Xu.

**Investigation:** Xinjuan Fu, Zhigang Xiu.

**Project administration:** Xinjuan Fu, Zhigang Xiu, Hongwei Xu.

**Resources:** Xinjuan Fu.

**Supervision:** Hongwei Xu.

**Writing – original draft:** Xinjuan Fu.

**Writing – review & editing:** Hongwei Xu.
